# Copper smelting slag valorization in waste-derived clay ceramics: sintering behavior, microstructural evolution, engineering performance, and leaching behavior

**DOI:** 10.1039/d6ra09416c

**Published:** 2026-10-30

**Authors:** Dias Tolegenov, Madeniyet Yelubay, Sabit Maussumbayev, Nurdana Kanasheva, Gulzat Aitkaliyeva, Tatyana Vakalova, Vladimir Mokichev, Sergey Volynkin, Anton Kasprzhitskii, Georgy Lazorenko

**Affiliations:** a Department of Chemistry and Chemical Technology, Toraighyrov University Pavlodar 140000 Kazakhstan diastolegenov1992@gmail.com Yelubay.m@tou.edu.kz mausumbaevsabit@gmail.com kanashevanur94@gmail.com; b Department of Chemical and Biochemical Engineering, Satbayev University Almaty 050013 Kazakhstan g.aitkaliyeva@satbayev.university; c N. M. Kizhner Scientific and Educational Center, Tomsk Polytechnic University Lenin Avenue, 30 Tomsk 634050 Russian Federation tvv@tpu.ru; d Climate Center, Novosibirsk State University Pirogov Street, 2 Novosibirsk 630090 Russia v.mokichev@g.nsu.ru volynkin_sergey@vk.com a.kasprzhitskii@nsu.ru g.lazorenko@nsu.ru

## Abstract

The Fe- and Si-rich composition of copper smelting slag (CS) provides a basis for its utilization as a secondary raw material in high-temperature ceramic processing. This study investigates the effect of relatively low additions of untreated CS (5 and 10 wt%) on the sintering behavior, phase and microstructural evolution, engineering properties, and leaching behavior of waste-derived ceramics fired at 900–1200 °C. The effect of CS was strongly temperature-dependent, with no clear mechanical benefit at 900–1000 °C but pronounced improvement at 1100–1200 °C. At 1200 °C, 10 wt% CS increased compressive strength to approximately 135 MPa and bulk density to 2.23 g cm^−3^, while reducing water absorption to 2.2%. XRD, FTIR, and SEM analyses indicated high-temperature structural reorganization and enhanced matrix densification without major changes in the dominant crystalline phase assemblage. CS incorporation did not systematically increase the release of potentially hazardous elements, with regulated concentrations remaining well below the corresponding TCLP limits. The results show that the partial replacement of SS and FA by low additions of untreated CS can improve ceramic performance while maintaining low leachability, supporting its valorization in high-performance ceramic materials. These findings provide a basis for further studies on the long-term durability and environmental performance of CS-containing ceramics under real service conditions, as well as for scaling-up toward industrial production.

## Abstract 2

Low additions of untreated copper smelting slag improve ceramic densification and performance while maintaining low leachability of hazardous elements.

## Graphical abstract

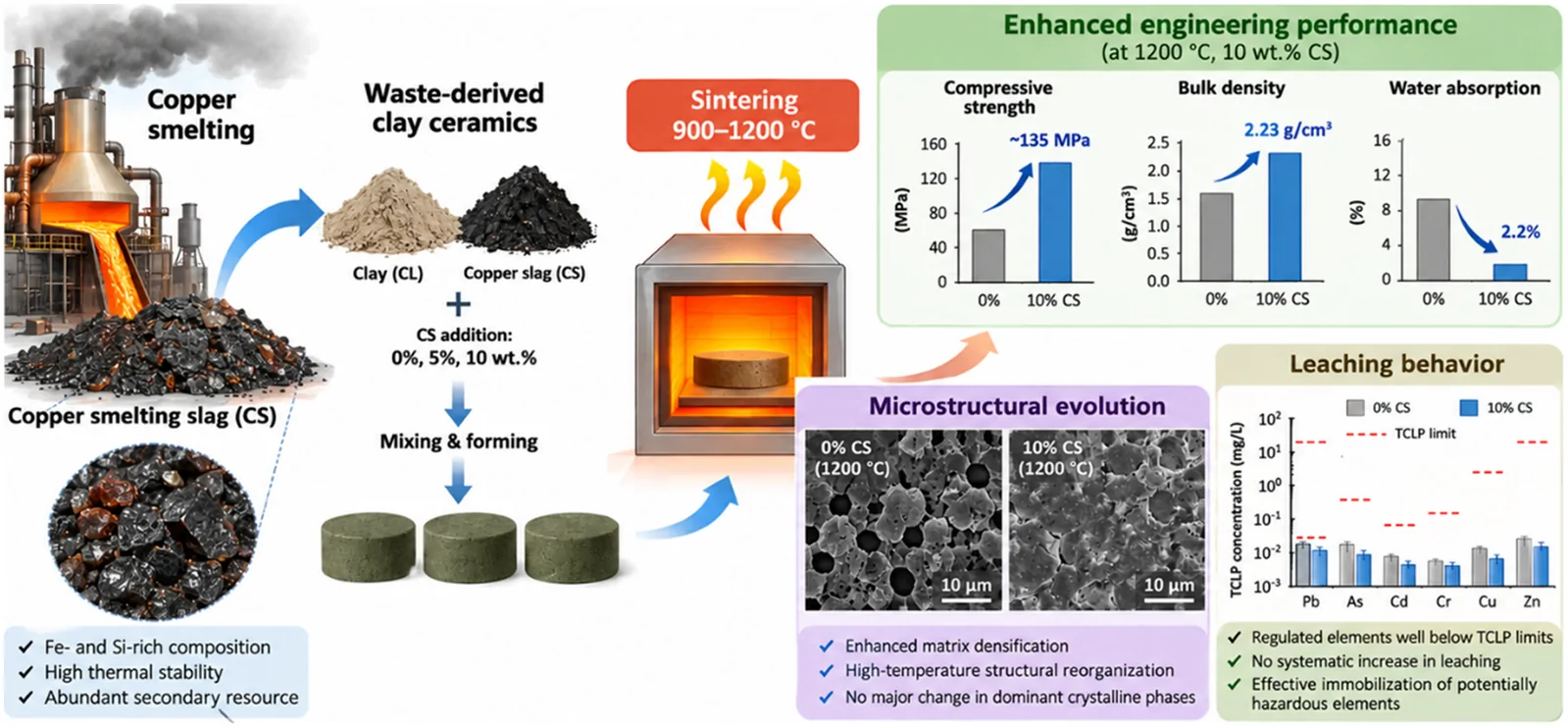

## 1 Introduction

The growing generation of metallurgical residues, coupled with the increasing demand for primary mineral resources, has intensified interest in converting industrial solid wastes into functional raw materials for construction and ceramic products.^1–3^ Among these residues, copper smelting slag (CS) represents a particularly challenging yet potentially valuable secondary resource.^4,5^ Its global generation is estimated at 24.6–37.7 million tonnes annually, highlighting the need for effective strategies for its large-scale valorization.^6^ CS is a by-product generated during the pyrometallurgical production of copper, where copper concentrates are smelted at high temperatures to separate the copper-rich matte from the silicate-rich slag phase (Fig. 1).^7,8^ CS is typically a dense, dark-colored material with a relatively high density (approximately 3.8 g cm^−3^) and hardness (approximately 7 on the Mohs scale),^9^ whose chemical and mineralogical composition varies with the concentrate, smelting technology, and process additives, although Fe- and Si-bearing phases generally predominate.^10,11^ The combination of abundant silicate and iron-bearing constituents with favorable physical characteristics makes CS a promising secondary raw material for high-temperature applications, particularly ceramic processing. However, the long-term disposal of CS may pose environmental concerns due to the presence of residual metals and potentially mobile trace elements. Adilov *et al.*^12^ reported substantial geochemical changes in soils affected by long-term copper slag disposal, including acidification to pH 3.5–4.0 and enhanced mobility of potentially hazardous elements. More than 6% of Cu, 3% of Zn, and 2% of Pb were found in mobile forms, while the water-soluble fractions exceeded 0.5% for Cu, 4% for Zn, 0.5% for As, and 1% for Pb. These observations reinforce the need for valorization routes that not only utilize CS but also ensure effective immobilization of potentially hazardous elements in the resulting products. This concern is further supported by Gabasiane *et al.*,^13^ who identified prolonged exposure of stockpiled CS to rainfall and weathering as a potential pathway for the release of Pb, As, Cd, Fe, Cu, and Co, with subsequent contamination of soils and water resources and reported field evidence of such effects in the Selebi-Phikwe mining area (Botswana). Importantly, the environmental mobility of metal(loid)s is governed not only by their total concentrations but also by their occurrence within individual slag phases. Tan *et al.*^14^ demonstrated that changes in the phase distribution of Cu and As markedly affected their leaching behavior. For a CS containing 4.38 wt% Cu and 0.19 wt% As, Cu and As concentrations in TCLP leachates varied from 22.2 to 78.6 mg L^−1^ and from 1.04 to 2.23 mg L^−1^, respectively, depending on cooling conditions and the resulting phase assemblage. The comparatively stable Cu matte exhibited limited dissolution, whereas Cu–As phases were considerably more susceptible to leaching; as was also identified in chemically dissolved form within the glassy silicate matrix.^14^ These findings underscore the importance of considering metal(loid) stabilization alongside material performance when developing high-temperature routes for CS valorization.

**Fig. 1:**
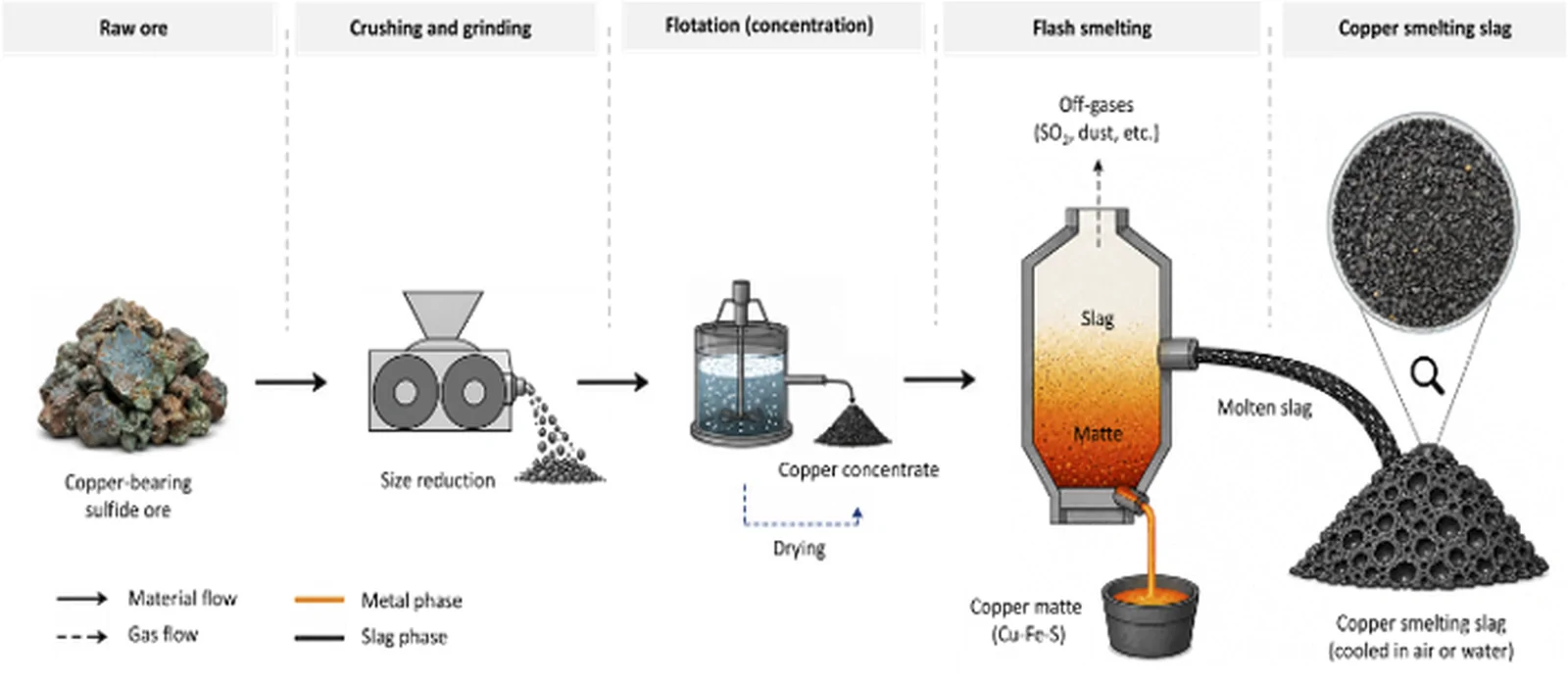
Schematic representation of copper smelting slag generation from copper-bearing sulfide ore.

A variety of routes have been investigated for CS valorization, ranging from recovery of residual metals to its incorporation into cementitious,^15–17^ aggregate,^18–20^ glass,^21–23^ and ceramic products.^24–26^ Ceramic processing is particularly attractive because it combines waste incorporation with high-temperature transformations capable of converting the original slag phases into a new, consolidated mineral matrix. Owing to its Fe- and Si-rich composition, CS can actively influence the firing process, promoting changes in phase assemblage, liquid-phase formation, densification, and pore structure that ultimately determine the engineering performance of the ceramic body. Previous studies have demonstrated the potential of CS as a raw material for sintered ceramics. Lemougna *et al.*^27^ obtained a compressive strength of up to 140 MPa for ceramics containing 50 wt% CS fired at 1100 °C, attributing densification to partial melting. However, further heating to 1150 °C promoted closed porosity and reduced strength. Hou *et al.*^28^ produced black ceramics from CS and chromium slag with a compressive strength of 127.2 MPa at 1200 °C, demonstrating the participation of Fe-bearing slag components in high-temperature phase formation. Liu *et al.*^29^ showed that ceramics containing 40 wt% reduced CS reached a flexural strength of 81.7 MPa and near-zero water absorption after modification with 4 wt% TiO_2_, although excessive liquid formation at higher TiO_2_ contents caused pore growth and property deterioration. Jordán *et al.*^30^ showed that replacing clay with up to 35 wt% CS, combined with small additions of MSW fly ash, progressively reduced water absorption and increased flexural strength; at a total waste content of 40 wt%, water absorption decreased to approximately 8% and bending strength reached approximately 18 MPa at 1050 °C. The firing process also promoted the immobilization of potentially hazardous elements, with Pb concentrations below 0.05 mg L^−1^ at 1000–1050 °C. Hou *et al.*^31^ further demonstrated the reactive role of Fe-rich CS in multislags ceramics, where high-temperature reactions produced Fe-bearing spinel phases; the optimized material reached a compressive strength of 162.61 MPa at 1150 °C, while Cr(vi) leaching decreased from 11.59 to 0.97 mg L^−1^. However, previous studies have predominantly employed relatively high CS contents, additional wastes or fluxing agents, or pretreated CS. The influence of small additions of untreated CS on phase evolution, densification, engineering properties, and leaching behavior over a broad firing-temperature range remains insufficiently understood.

Accordingly, this work investigates the incorporation of copper smelting slag into waste-derived clay ceramics containing coal fly ash and steel slag, with particular emphasis on the coupled effects of CS content and firing temperature. The evolution of the material across the investigated firing range is examined through phase, bonding, and microstructural characterization and related to densification and key engineering properties, including compressive strength, bulk density, volumetric shrinkage, and water absorption. Leaching behavior is additionally evaluated to assess the environmental implications of incorporating CS into the fired ceramic matrix. Particular attention is given to whether CS, even at relatively low contents, can act as a functionally active component rather than merely waste filler by influencing high temperature transformations, microstructural development, and the resulting engineering and environmental performance. By establishing relationships between compositions, sintering behavior, microstructure, and properties, this study seeks to clarify the role of CS in waste-containing clay ceramic systems and assess its potential for effective valorization through ceramic processing.

## 2 Experimental program

### 2.1 Materials

Copper slag (CS) was obtained commercially from Uralgrit (Russia) as an abrasive-grade powder produced at the Rezh Abrasive Plant (Rezh, Sverdlovsk Region, Russia) from processed copper smelting slag recovered from historical dumps. While not a primary smelting slag, this material is representative of commercially processed historical CS and is therefore relevant to the valorization of stockpiled slag deposits. Refractory clay (CL) from the Kemertuz deposit (Pavlodar Region, Kazakhstan) was used as the clayey component. Steel slag (SS) was supplied by KSP Steel (Pavlodar, Kazakhstan), while coal fly ash (FA), generated from the combustion of Ekibastuz coal, was collected from thermal power plants in Pavlodar, Kazakhstan. Prior to processing, all raw materials were oven-dried at 100 °C until a constant mass was reached (UT-4603, Xieli International Trading Co., China). The dried materials were then ground for 5 min at 400 rpm using a planetary ball mill (BM6 Pro, POWTEQ, China) equipped with zirconia jars and milling media. After grinding, the powders were passed through a 0.05 mm sieve to obtain the required particle size fraction. The characteristics of CL, FA, and SS have been reported previously^32–34^ and are summarized here together with those of CS. The morphology and elemental composition of the raw materials were examined using a TESCAN MIRA scanning electron microscope (TESCAN, Czech Republic) equipped with an energy-dispersive X-ray spectroscopy (EDS) system. SEM imaging was performed at an accelerating voltage of 20 kV and a beam current of 1400 pA, and representative micrographs and EDS spectra are presented in Fig. 2. The raw materials exhibited markedly different particle morphologies, including angular, platy, irregular, and agglomerated particles, consistent with their different origins. EDS analysis revealed Si and Al as major constituents in the aluminosilicate raw materials, whereas the relative abundance of Fe, Ca, Mg, and alkali elements varied considerably among the investigated materials.^35^

**Fig. 2:**
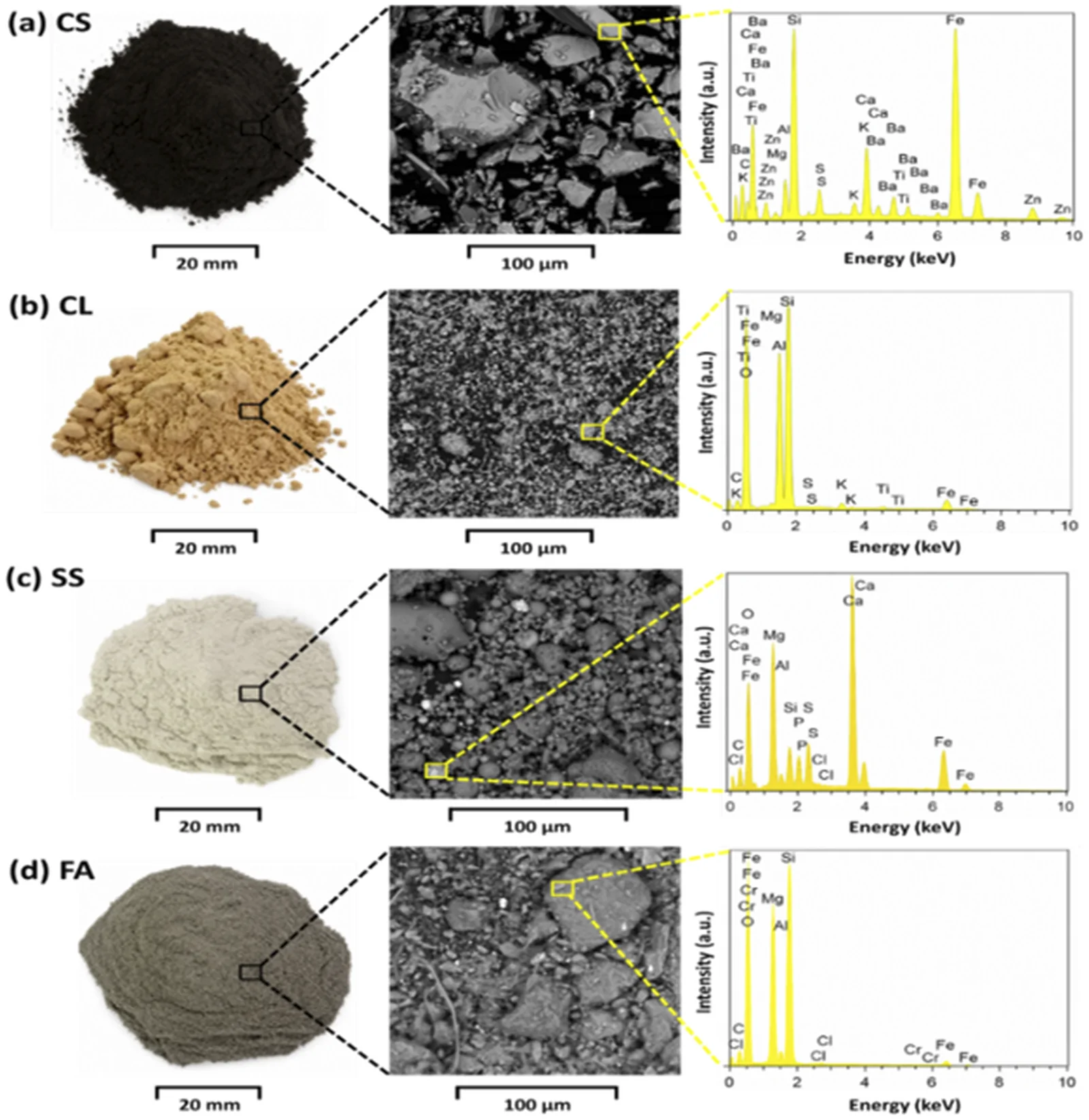
Optical macrographs, SEM images and point EDS spectrum of raw materials: (a) CS, (b) CL, (c) SS and (d) FA.

The bulk chemical composition of the raw materials was determined by X-ray fluorescence (XRF) spectroscopy using an ARL 9900 XP spectrometer (Thermo Electron Corporation, USA). Prior to analysis, the dried powders were calcined at 1000 °C for 2.5 h and subsequently fused with a lithium borate flux consisting of 66.67 wt% lithium tetraborate, 32.83 wt% lithium metaborate, and 0.50 wt% lithium bromide. A sample-to-flux mass ratio of 1 : 9 was used, and fusion was performed in platinum crucibles using a Lifumat-2.0-Ox automatic fusion system (Linn High Therm, Germany). The resulting homogeneous glass beads were used for XRF measurements. As shown in Table 1, the raw materials differed markedly in their chemical composition, determined by XRF and ICP-AES methods. CL and FA were predominantly aluminosilicate materials, containing 58.7 and 51.9 wt% SiO_2_ and 21.9 and 26.0 wt% Al_2_O_3_, respectively.

Chemical compositions of the raw materials determined

**Table:** 

Major composition	Mass percentage (%)			
	CL	SS	FA	CS
Silicon dioxide	58.7	18.7	51.9	35.4
Aluminum oxide	21.9	13.3	26.0	7.9
Iron oxide	3.7	0.3	11.8	42.1
Calcium oxide	1.3	60.4	2.1	7.8
Magnesium oxide	0.5	6.0	0.8	1.5
Sodium oxide	0.3	0.1	0.5	0.3
Titanium oxide	1.5	0.2	1.1	0.3
Others	12.1	1.0	5.8	4.7

**Table:** 

Minor composition	mg kg^−1^			
	CL	SS	FA	CS
Cr	33	<5.0	5.7	390
Mn	12	<10	<10	2500
Ni	8.9	0.6	1.4	30
Cu	<5.0	15	7.4	2700
Zn	23	70	1.5	2700
As	9.2	3.1	18	230
Cd	2.9	0.9	1.0	5.0
Pb	40	29	<10	580

SS was distinguished by its high CaO content (60.4 wt%), which is reflected in the predominance of Ca-bearing phases, mayenite and Ca-olivine, identified by XRD (Table 2), whereas CS exhibited a distinctly Fe–Si-rich composition, with Fe oxide and SiO_2_ contents of 42.1 and 35.4 wt%, respectively. CS also contained 7.9 wt% Al_2_O_3_ and 7.8 wt% CaO, while the remaining individual oxides occurred at considerably lower concentrations.

**Table 2:** Phase composition of raw materials

Major composition	Mass percentage (%)			
	CL	SS	FA	CS
Mullite	—	—	67.5	—
Quartz	9.3	—	19.8	—
Magnetite	—	—	2.7	—
Glassy phase	—	—	10.0	100
Ca-olivine	—	38.7	—	—
Mayenite	—	40.7	—	—
Jasmundite	—	8.7	—	—
Periclase	—	3.1	—	—
Wollastonite	—	3.4	—	—
Enstatite	—	2.6	—	—
Corundum	—	2.3	—	—
Pyrite	—	0.4	—	—
Pyrrhotite	—	0.1	—	—
Montmorillonite	25.3	—	—	—
Kaolinite	38.5	—	—	—
Chlorite	8.1	—	—	—
Gypsum	5.2	—	—	—
Illite	13.6	—	—	—

The bonding characteristics of the raw materials were further examined by Fourier-transform infrared (FTIR) spectroscopy (Fig. 3b). Spectra were recorded using an FT-801 spectrometer (Simex, Russia) equipped with a single-reflection attenuated total reflectance (ATR) accessory with a ZnSe crystal. Measurements were performed at a spectral resolution of 4 cm^−1^, with 50 scans averaged for each spectrum. The spectra exhibited distinct features consistent with the different chemical and mineralogical nature of the raw materials. CL showed characteristic Si–O vibrations together with O–H stretching and deformation bands associated with clay minerals. FA exhibited vibrations attributable mainly to Si–O–Si and Si–O–Al environments, while the SS spectrum was dominated by Si–O vibrations. CS displayed a broad intense Si–O stretching band, consistent with its predominantly silicate glassy structure, together with a weaker carbonate-related feature.

**Fig. 3:**
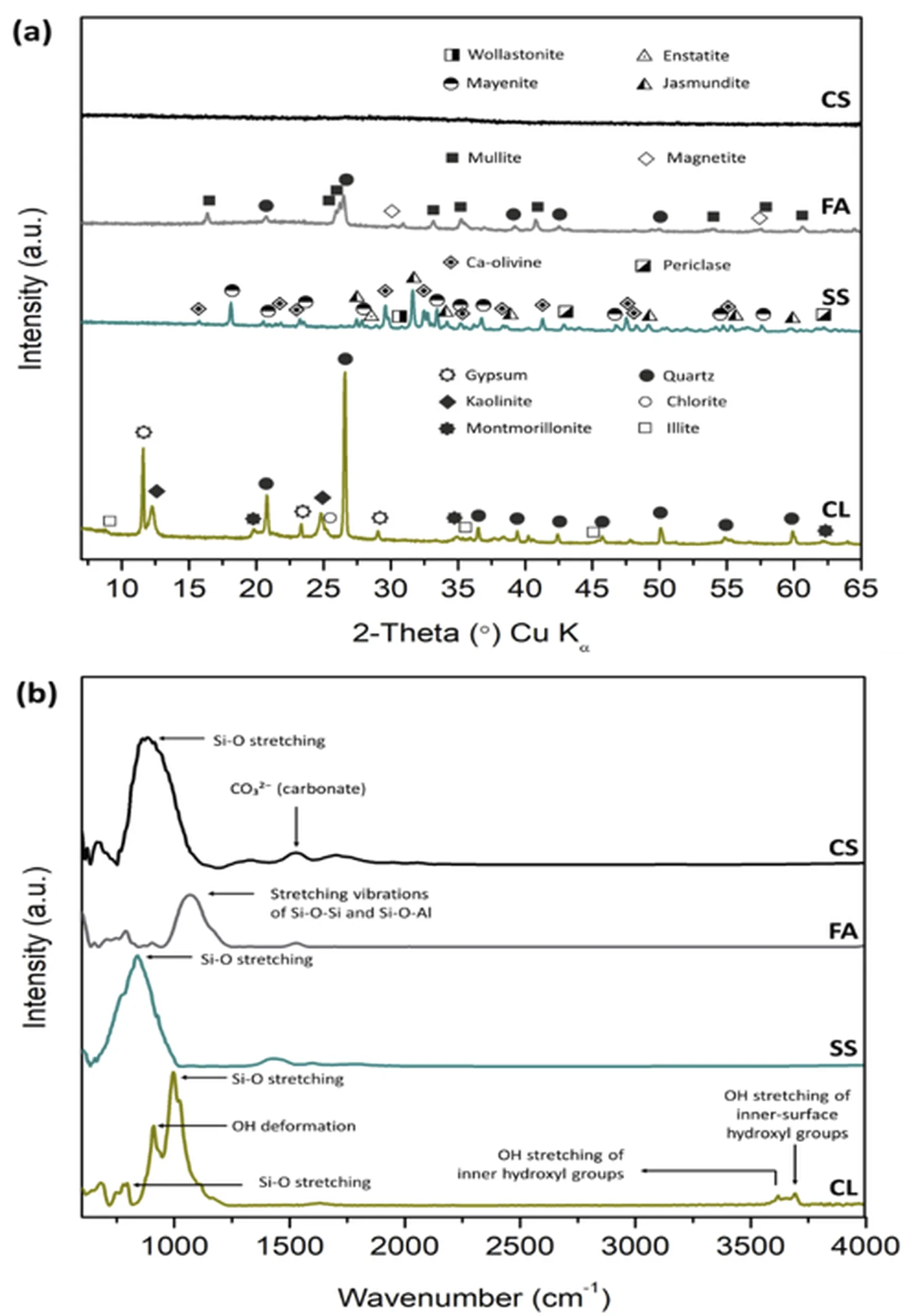
(a) XRD patterns and (b) FTIR spectra for raw materials.

The crystalline and amorphous phase assemblages were determined by X-ray diffraction (XRD) using a DRON-8 diffractometer (Burevestnik, Russia) with Cu-Kα radiation (*λ* = 1.5406 Å). Diffraction patterns were collected over a 2*θ* range of 6–65° with a step size of 0.05° (Fig. 3a). The quantitative phase composition is summarized in Table 2. CS was entirely represented by a glassy phase, confirming its predominantly amorphous character. FA consisted mainly of mullite (67.5 wt%) and quartz (19.8 wt%), together with 10.0 wt% glassy phase and 2.7 wt% magnetite.

SS exhibited a markedly different mineralogy dominated by mayenite (40.7 wt%) and Ca-olivine (38.7 wt%), with smaller amounts of jasmundite (8.7 wt%), wollastonite (3.4 wt%), periclase (3.1 wt%), enstatite (2.6 wt%), corundum (2.3 wt%), pyrite (0.4 wt%), and pyrrhotite (0.1 wt%).^36^ CL showed a heterogeneous clay-mineral assemblage comprising kaolinite (38.5 wt%), montmorillonite (25.3 wt%), illite (13.6 wt%), quartz (9.3 wt%), chlorite (8.1 wt%), and gypsum (5.2 wt%).

### 2.2 Preparation process

The raw materials (CL, FA, SS, and CS) were proportioned according to the formulations given in Table 3. The dry components of each formulation were homogenized in a blade mill at 1000.00 rpm for 1 min. Subsequently, 100 g of each homogenized mixture was combined with 15 mL of deionized water and mixed for 10 min. Green specimens were formed by uniaxial pressing of 50 g portions of the moistened mixture in a steel mold under a pressure of 10 MPa for 60 s, with the pressure increased linearly and automatically in increments of 0.3 MPa. The compacted specimens were then dried at 60 °C for 18 h. Two specimen geometries were prepared depending on the subsequent characterization: discs with a diameter of 25 mm for water absorption, volume shrinkage, bulk density, and structural analyses, and cylindrical specimens (25 mm in diameter and 50 mm in height) for compressive strength measurements. Sintering was performed in an LF-15/13-V11 muffle furnace (LOIP, Russia) at 900, 1000, 1100, or 1200 °C. Firing was carried out under ambient atmospheric conditions, without vacuum. After reaching the final firing temperature, the specimens were heated from room temperature to the target temperature at 3 °C min^−1^, held for 6 h, and subsequently allowed to cool inside the furnace until the temperature reached 100 °C. The specimens were then removed from the muffle furnace and allowed to cool to room temperature. At least five specimens were prepared for each composition and firing temperature. The overall preparation and characterization procedure is summarized in Fig. 4.

**Table 3:** Ratio of raw materials for different experimental formulations

Formulations	CL (wt%)	SS (wt%)	CFA (wt%)	CS (wt%)	Firing conditions	
					Temperature (°C)	Time (h)
CS0-900	80	10	10	0	900	6
CS0-1000	80	10	10	0	1000	6
CS0-1100	80	10	10	0	1100	6
CS0-1200	80	10	10	0	1200	6
CS5-900	80	7.5	7.5	5	900	6
CS5-1000	80	7.5	7.5	5	1000	6
CS5-1100	80	7.5	7.5	5	1100	6
CS5-1200	80	7.5	7.5	5	1200	6
CS10-900	80	5	5	10	900	6
CS10-1000	80	5	5	10	1000	6
CS10-1100	80	5	5	10	1100	6
CS10-1200	80	5	5	10	1200	6

**Fig. 4:**
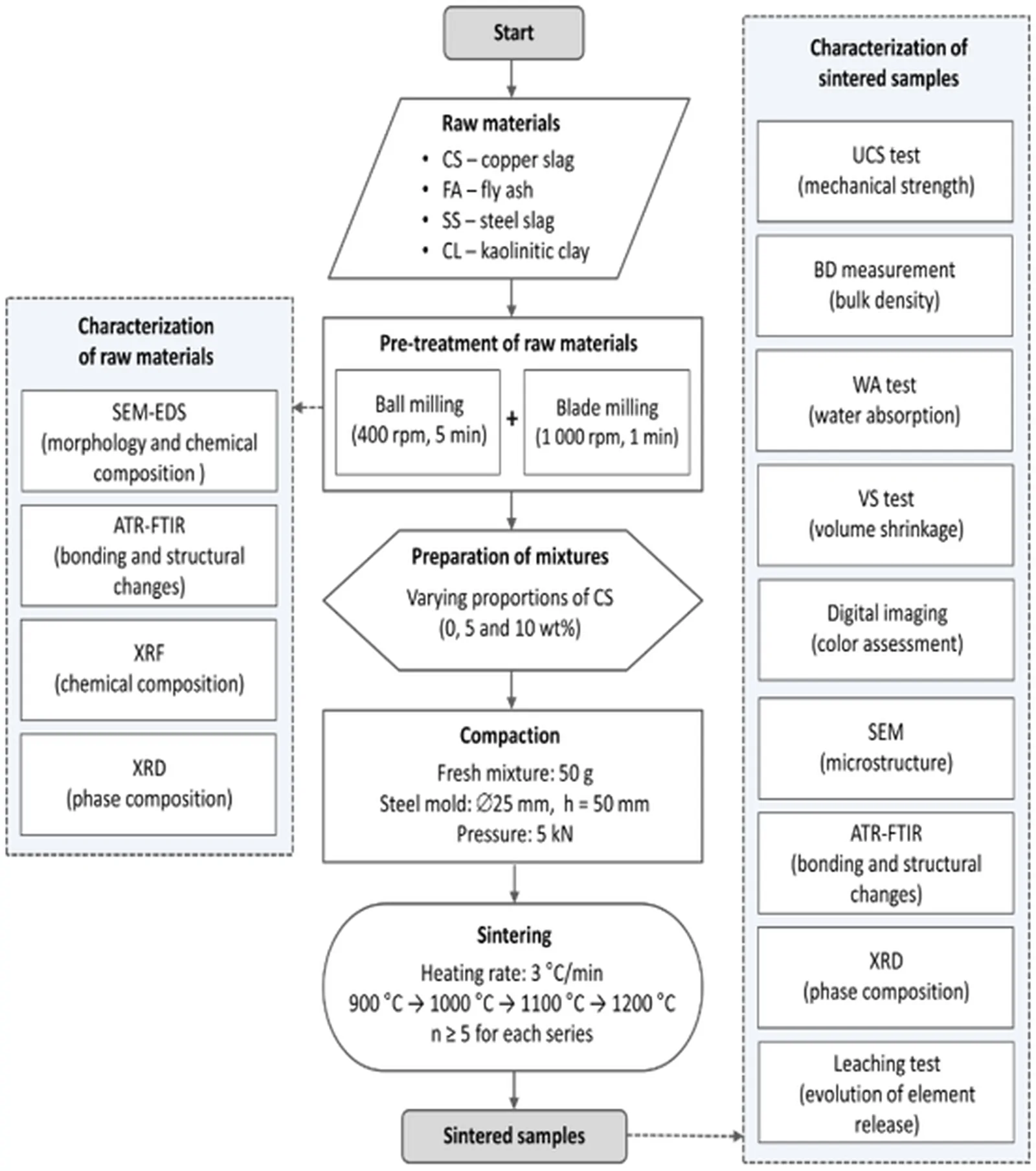
Experimental workflow for ceramic preparation and characterization.

### 2.3 Test methods

Structural changes induced by firing were examined by ATR-FTIR spectroscopy and X-ray diffraction. ATR-FTIR spectra were collected using an FT-801 spectrometer (NPF SIMEX, Russia) equipped with a ZnSe ATR crystal over the range of 600–4000 cm^−1^ at a spectral resolution of 4 cm^−1^. At least 50 scans were averaged for each spectrum. A background spectrum of the cleaned ATR crystal was recorded before each measurement, after which the powdered sample was brought into firm contact with the crystal. The ATR crystal was cleaned with ethanol between measurements.

XRD patterns of the sintered specimens were recorded using a DRON-8 diffractometer (Burevestnik, Russia) with Cu-Kα radiation (*λ* = 1.5406 Å) over a 2*θ* range of 6–65°, using a step size of 0.05° and a counting time of 3 s per step.^36^ Quantitative phase analysis was performed by the Rietveld method using the “Method Rietveld – Rietveld” software.^37^ The microstructure of the sintered ceramics was examined on freshly fractured surfaces using a Hitachi S-3400N scanning electron microscope operated in backscattered-electron (BSE) mode at an accelerating voltage of 20 kV. The unconfined compressive strength (UCS) of the cylindrical specimens was measured using an IP-100 universal testing machine (ZIPO, Russia) equipped with a 100 kN load cell. Loading was applied continuously at a rate of 0.5 MPa s^−1^ until failure. UCS was calculated as:1
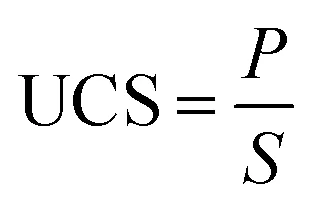
where *P* is the maximum load at failure (MN) and *S* is the initial cross-sectional area of the specimen (m^2^). The mechanical strength tests were conducted using a procedure based on GOST R 58527-2023.^38^ All measurements were performed on at least five independent specimens (*n* = 5) per composition and firing temperature; results are reported as mean ± standard deviation.

Water absorption (WA) was determined by immersing the fired specimens in deionized water for 24 h under ambient laboratory conditions (23 ± 2 °C and 65 ± 5% relative humidity), following the general principles of GOST 7025-91,^39^ with modifications to the immersion time. After immersion, the specimens were removed from the water, surface water was removed, and the saturated mass was recorded. WA was calculated as:2
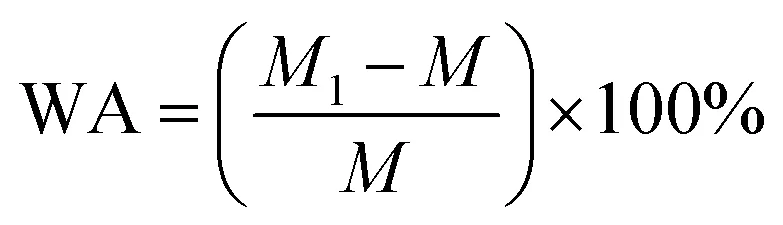
where *M* is the dry mass of the fired specimen (g) and *M*_1_ is its water-saturated mass after 24 h immersion (g).

Bulk density (BD) was determined based on the measured mass and geometric dimensions of the fired specimens. Mass measurements were performed using a BEL DA-1203C analytical balance (BEL Engineering, Italy) with a maximum capacity of 1200 g and a readability of 0.001 g. BD was calculated according to eqn (3):3
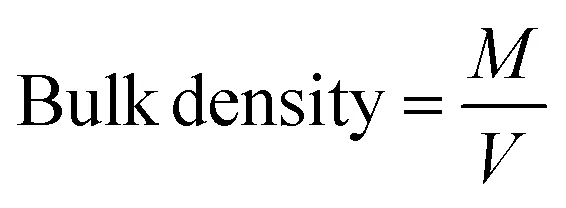
where *M* is its dry mass after firing (g) and *V* is its geometric volume.

Volume shrinkage (VS) was determined from the change in specimen dimensions during firing. The diameter and height of each specimen were measured before and after sintering, and the corresponding geometric volumes were calculated assuming a cylindrical shape. VS was expressed as the percentage reduction in volume relative to the initial volume of the dried green specimen:4
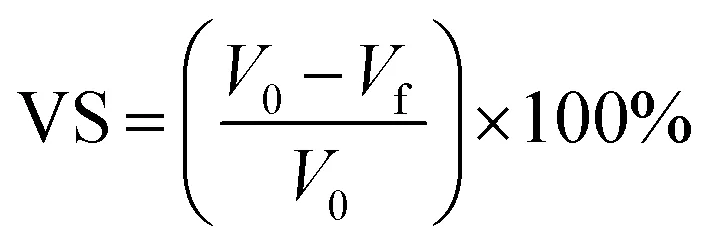
where *V*_0_ is the geometric volume of the dried green specimen before firing (cm^3^) and *V*_f_ is the geometric volume of the specimen after firing (cm^3^).

Digital images of the fired specimens were acquired for comparative assessment of their surface color and appearance. Prior to imaging, the specimen surfaces were ground using a water-cooled diamond disc to remove surface contamination, followed by dust removal and drying. The prepared surfaces were scanned using an Epson Perfection V600 flatbed scanner (Epson, Japan) with Epson Scan software under identical standard settings. Images were saved in TIFF format.

The leaching of heavy metals from the ceramic specimens and the original raw materials was evaluated using the EPA Method 1311 (Toxicity Characteristic Leaching Procedure, TCLP).^40^ The ceramic specimens were crushed to a particle size of 1–10 mm, and particles smaller than 1 mm were removed by sieving. The extraction fluid was selected in accordance with Section 7.1.4 of EPA Method 1311. For the ceramic samples, following the Section 7.1.4 procedure, the pH after acid treatment was below 5.0; therefore, Extraction Fluid No. 1 (acetate buffer, pH 4.93) was used. A 50 g portion of each sample was mixed with the extraction solution at a solid-to-liquid (S/L) ratio of 1 : 20 and agitated for 18 h. Following extraction, the leachates were centrifuged and acidified with concentrated nitric acid. The total heavy metal contents of the original raw materials were determined following microwave-assisted digestion. Heavy metal concentrations in the digests and leachates were measured by inductively coupled plasma optical emission spectrometry (ICP-OES) using an iCAP 6500 Duo spectrometer (Thermo Scientific, USA) in its standard configuration. Matrix effects were corrected using an internal standard method with scandium nitrate solution as the internal standard. The detection limits for Cr, Mn, Ni, Cu, Zn, As, Cd and Pb were 1.0, 20, 4.0, 1.0, 1.3, 10, 0.2, 10 µg L^−1^, respectively. All measurements were performed in triplicate, and the relative standard deviation (RSD) of the results did not exceed 15%. Calibration was carried out using certified multielement standard solutions (MES; NPP Skat LLC, Russia). High-purity argon (99.95%) was used as the plasma, carrier, and cooling gas.

## 3 Results and discussion

### 3.1 Physical indicators and mechanical properties

The physical and mechanical properties of the ceramics were strongly dependent on both firing temperature and CS content (Fig. 5). In all compositions, increasing the firing temperature from 900 to 1200 °C resulted in a substantial increase in compressive strength and bulk density, accompanied by a pronounced decrease in water absorption. These trends indicate progressive consolidation and densification of the ceramic matrix during firing.

**Fig. 5:**
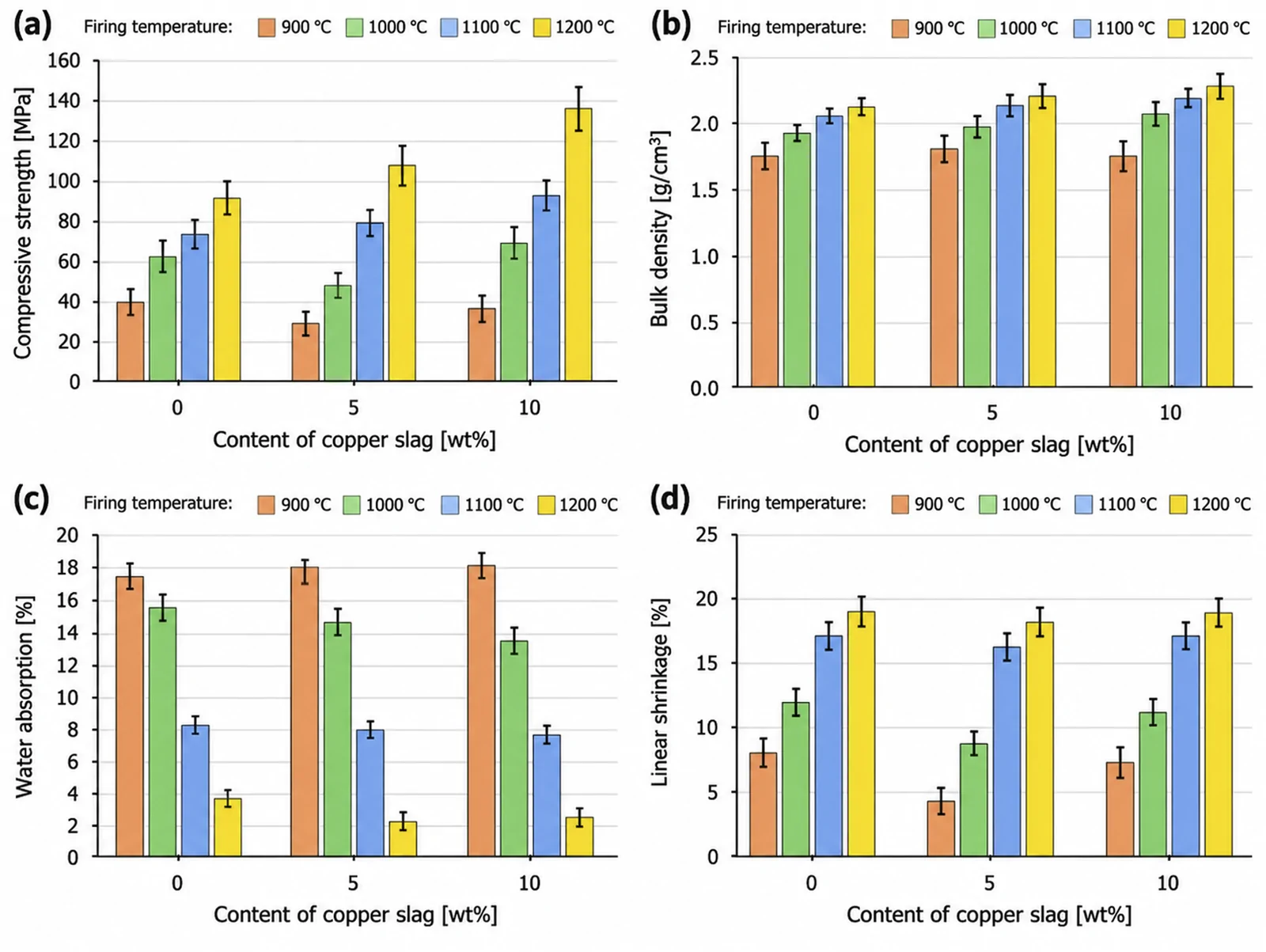
Evolution of compressive strength (a), bulk density (b), water absorption (c) and volume shrinkage (d) of the prepared ceramics as a function of copper slag addition and firing temperature. Error bars represent standard deviation (*n* = 5).

The effect of CS addition was more complex and depended strongly on the firing temperature. The compressive strength increased markedly with temperature for all compositions (Fig. 5a). The CS-free specimens exhibited an increase from approximately 40 MPa at 900 °C to about 91 MPa at 1200 °C. At 5 wt% CS, the corresponding values were approximately 30 and 108 MPa, while the addition of 10 wt% CS resulted in an increase from about 36 MPa at 900 °C to 135 MPa at 1200 °C. Thus, CS addition did not improve the strength at the lowest firing temperature; at 900 °C, both CS-containing compositions showed lower values than the reference ceramic. A similar tendency persisted at 1000 °C, particularly for the 5 wt% CS composition. At higher temperatures, however, the effect changed considerably. At 1100 °C, the strength of CS5 and CS10 reached approximately 79 and 91 MPa, respectively, compared with about 74 MPa for CS0. The difference became most pronounced at 1200 °C, where 5 and 10 wt% CS increased compressive strength by approximately 19 and 48%, respectively, relative to the CS-free composition. The maximum value of approximately 135 MPa was therefore achieved for CS10 fired at 1200 °C. This temperature-dependent behavior may be explained by the increasing involvement of CS in high-temperature sintering through the formation of a liquid phase, enhanced particle bonding, and progressive matrix densification, as suggested in previous studies.^26,27^ Lin *et al.*^26^ similarly associated improved mechanical performance of CS containing ceramics at elevated temperatures with intensified sintering and reduced porosity, while Lemougna *et al.*^27^ reported progressive densification and an increasing amorphous fraction with firing temperature. These observations are consistent with the pronounced strength enhancement obtained here at 1100–1200 °C, particularly for CS10. Quantitatively, the present CS10 ceramic (∼135 MPa at 1200 °C) is comparable to the ∼140 MPa reported by Lemougna *et al.*,^27^ but is obtained at a markedly lower CS content (10 wt%).

The evolution of bulk density generally followed the strength development (Fig. 5b). For CS0, bulk density increased from approximately 1.76 g cm^−3^ at 900 °C to 2.11 g cm^−3^ at 1200 °C. The CS containing specimens showed comparable temperature-dependent increases, reaching approximately 2.15 g cm^−3^ for CS5 and 2.23 g cm^−3^ for CS10 at 1200 °C. At 900 °C, the incorporation of CS had little systematic effect on density, whereas at 1000–1200 °C the density generally increased with CS content. The highest bulk density was obtained for CS10 at 1200 °C, coinciding with the highest compressive strength. This correspondence suggests that the beneficial effect of CS at elevated temperatures is associated, at least in part, with more effective consolidation of the ceramic body.^27,41^

Water absorption showed the opposite trend (Fig. 5c), decreasing sharply with increasing firing temperature. At 900 °C, all specimens exhibited relatively high and similar water absorption of approximately 18%. Increasing the temperature to 1000 °C reduced the values to approximately 14–16%, followed by a further decrease to about 8–9% at 1100 °C. After firing at 1200 °C, water absorption fell to approximately 2–3%. At each temperature above 900 °C, the CS containing specimens generally exhibited lower water absorption than CS0, with the effect becoming more evident as the firing temperature increased. At 1200 °C, water absorption decreased from approximately 3.1% for CS0 to about 2.4 and 2.3% for CS5 and CS10, respectively, although this difference is small and lies within the experimental scatter. The simultaneous increase in bulk density and decrease in water absorption indicate a reduction in accessible pore volume and increasingly effective densification at elevated firing temperatures.^26,27,42^

The dimensional changes during firing provide further evidence of this process (Fig. 5d). Volume shrinkage increased substantially with firing temperature for all compositions, reaching approximately 18–19% at 1200 °C. Unlike bulk density and water absorption, however, shrinkage did not vary monotonically with CS content. The CS5 specimens consistently exhibited the lowest shrinkage, particularly at 900 °C, whereas CS10 showed values closer to those of the reference composition. At 1200 °C, the differences among the compositions became relatively small despite the pronounced differences in compressive strength. The simultaneous evolution of these properties suggests that CS increasingly contributes to high-temperature densification rather than acting merely as an inert filler.

The macroscopic appearance of the ceramics also changed systematically with firing temperature and CS content (Fig. 6). At 900 °C, all compositions exhibited relatively similar light reddish-brown surfaces, although the CS containing specimens showed a slightly darker appearance. With increasing firing temperature, the differences became progressively more pronounced. While the CS free ceramic remained comparatively light even at 1200 °C, the addition of 5 and 10 wt% CS resulted in substantially darker brown–gray surfaces, particularly at 1100–1200 °C. The strongest visual contrast was therefore observed at the highest firing temperature. The progressive darkening with increasing CS content is consistent with the high Fe content of CS and temperature-induced transformations of Fe-bearing species during firing. Iron oxides and Fe-containing crystalline or glassy phases are known to exert a strong influence on the coloration of fired ceramics, while their oxidation state and incorporation into newly formed phases depend on firing conditions.^43–45^ The increasingly pronounced effect of CS at 1100–1200 °C is therefore consistent with its active participation in high-temperature transformations of the ceramic matrix rather than simple physical dilution of the original raw materials.

**Fig. 6:**
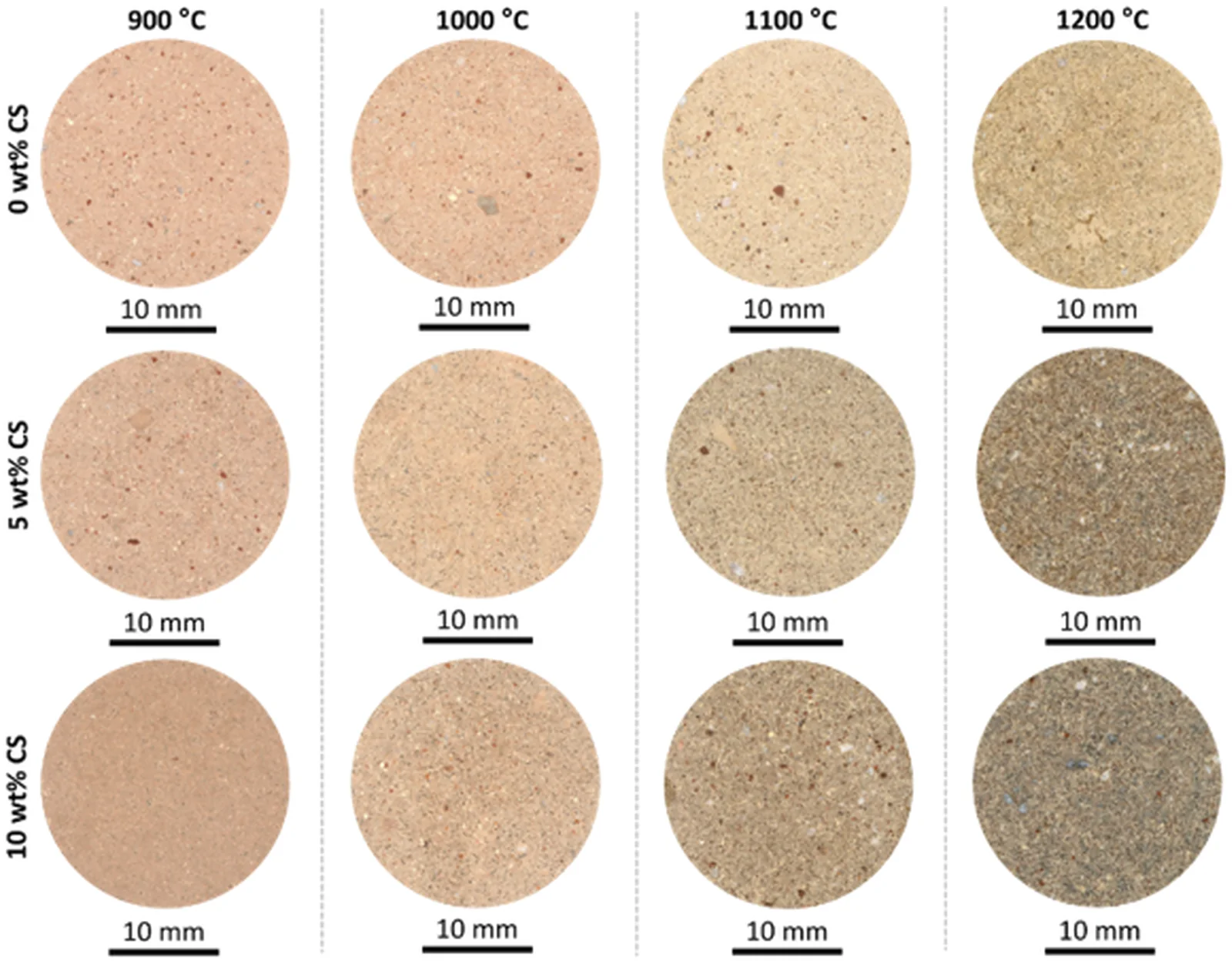
Visual appearance of ceramic samples with different CS contents sintered at different temperatures.

### 3.2 Phase composition and microstructure

The structural characterization was focused on the two end-point temperatures (900 and 1200 °C), corresponding to the states before and after the pronounced improvement in properties, while the intermediate stage is reflected in the property trends (Fig. 5). The XRD patterns reveal a pronounced effect of firing temperature on the phase assemblage, whereas variations in CS content produce comparatively smaller changes in the major crystalline phases (Fig. 7a). At 900 °C, the diffraction patterns of all compositions are dominated by quartz, accompanied by weaker reflections of mullite. No distinct additional crystalline phases attributable to CS are observed with increasing CS content. Considering the entirely glassy character of the initial CS determined by XRD (Table 2), its incorporation at 5–10 wt% therefore does not lead to the formation of substantial amounts of additional well crystallized phases detectable by XRD at this temperature. Increasing the firing temperature to 1200 °C results in a marked restructuring of the phase assemblage. Quartz and mullite remain detectable, while pronounced reflections assigned to cristobalite appear in all three compositions. The formation of cristobalite at 1200 °C and its absence at 900 °C should be considered primarily as a kinetic and phase-composition effect. Cristobalite in ceramics it often forms at 1000–1200 °C through intermediate stages involving the glassy phase, fluxes and amorphous silica.^46^ The formation of high temperature silica polymorphs during thermal treatment of Cu bearing slags and related ceramic systems has been reported previously.^26,41^ In particular, temperature-induced transformation of silica toward cristobalite has been observed during the thermal treatment of copper slag, while the phase evolution of CS containing ceramics has been shown to depend strongly on firing temperature and the chemical environment of the matrix.^26,47^ Despite the substantial differences in the physical and mechanical properties of CS0, CS5, and CS10 at 1200 °C, their XRD patterns remain qualitatively similar in terms of the major crystalline phases. Thus, the enhanced performance of CS containing ceramics at high temperatures appears to involve factors beyond changes in the major crystalline phase assemblage.

**Fig. 7:**
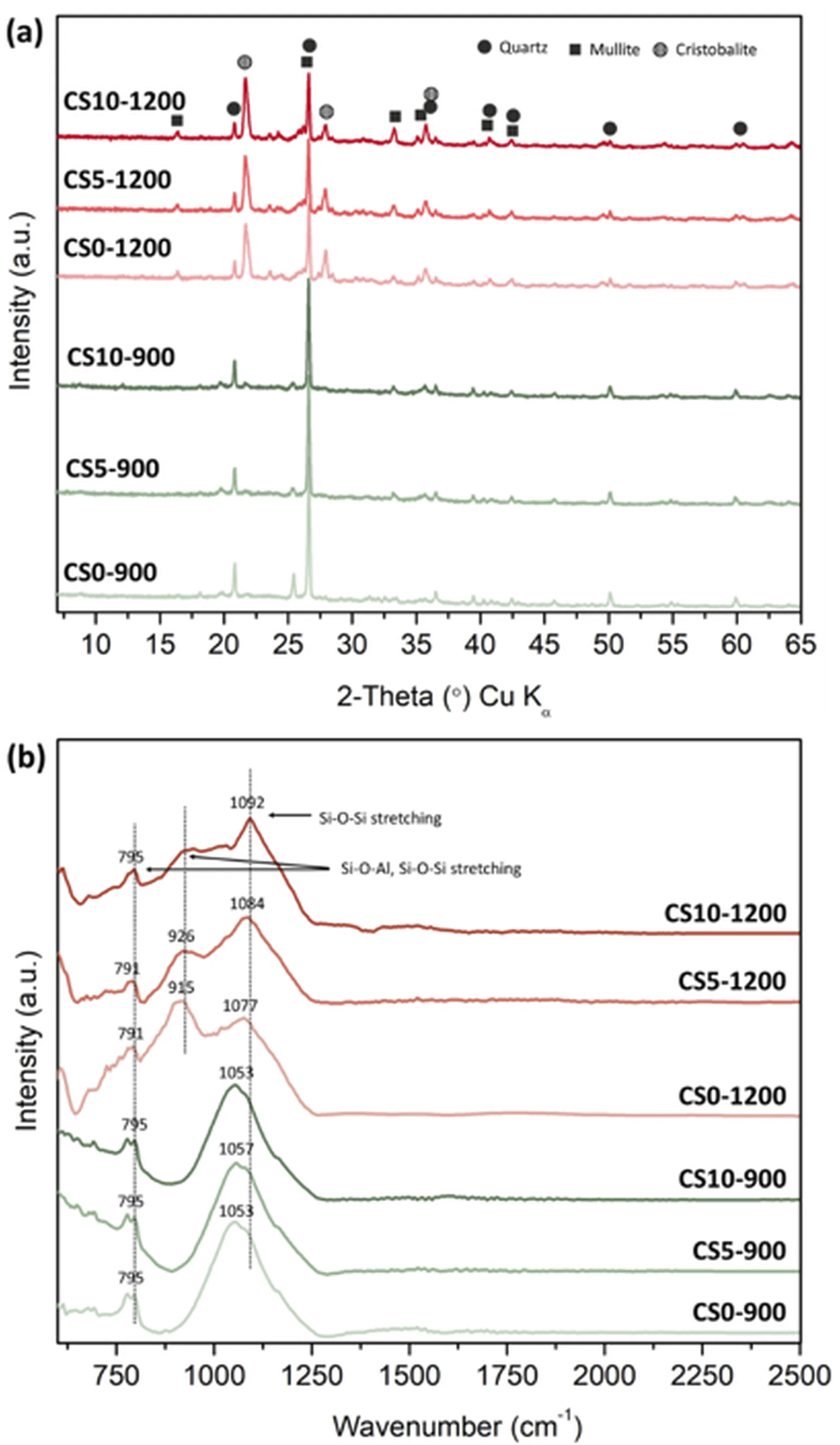
(a) XRD patterns and (b) FTIR spectrum for selected ceramic samples at different firing temperature (900 and 1200 °C) and CS dosage (0, 5 and 10 wt%).

Changes in the relative proportions of crystalline and non-crystalline constituents, together with enhanced high-temperature sintering, may instead contribute to the observed densification. This interpretation is consistent with previous studies in which CS promoted ceramic densification and increased the glass-phase fraction, with consequent improvements in mechanical properties.^26,27^

The FTIR spectra further demonstrate structural changes in the ceramic matrix during firing (Fig. 7b). At 900 °C, all compositions exhibit a broad dominant band centered at approximately 1053–1057 cm^−1^, together with a weaker feature near 795 cm^−1^. The broad absorption region between approximately 700 and 1200 cm^−1^ is characteristic of stretching vibrations within silicate structural units, while bands in this region are sensitive to the connectivity and local environment of SiO_4_ tetrahedra.^48,49^ Similar spectral features have been reported for silicate-rich copper slags, with the 700–1200 cm^−1^ region assigned predominantly to stretching vibrations of silicate tetrahedral units.^43^ After firing at 1200 °C, substantial changes occur in the shape and position of the main Si–O related absorption region. The effect is particularly evident for CS10-1200, where the dominant band shifts to approximately 1092 cm^−1^ and an additional feature appears near 926 cm^−1^. These spectral changes indicate modification of the local silicate/aluminosilicate bonding environment during high temperature treatment. The structure of CS derived silicate networks is known to be sensitive to network connectivity and the incorporation of network modifying and network forming cations, which can alter the distribution of Si–O–Si and Si–O–Al environments.^48,49^ The shift in the Si–O stretching vibration band from approximately 1053 to 1092 cm^−1^ during the firing of ceramics serves as a direct spectroscopic indicator of structural rearrangement within the silicates, reflecting the evolution of the ceramic matrix during the firing process. This process involves the transformation of the weakly polymerized structure of clay minerals into a more rigid, three-dimensionally linked network of aluminosilicate glass and newly formed crystalline phases. The XRD patterns reveal the presence of quartz and, in the specimens fired at 1200 °C, additional reflections assigned to mullite and cristobalite, indicating changes in the crystalline phase assemblage at higher temperatures. These findings provide complementary evidence that the structural evolution of the ceramic matrix may contribute to the observed FTIR changes.^50,51^^.^

The fracture surface micrographs provide direct evidence of the pronounced microstructural evolution with firing temperature (Fig. 8). At 900 °C, all compositions exhibit a relatively heterogeneous and weakly consolidated structure, with distinguishable residual particles, interparticle voids, and regions of incomplete sintering (Fig. 8a, c and e). The pore-area fraction, estimated using digital image processing, is highest for CS0-900 at 25–35% and remains comparably high for CS5-900 at 20–25% and CS10-900 at 20–30%, the porosity being dominated in all three cases by fine interconnected interparticle voids of roughly 2–15 µm. Although some local differences are observed among the compositions, the addition of CS does not result in substantial matrix consolidation at this temperature, since the apparent reduction in pore-area fraction relative to CS0-900 (about 20–25% for CS5-900 and 15–20% for CS10-900) is comparable to the uncertainty of the estimate. This observation is consistent with the limited effect of CS on the physical and mechanical properties at 900 °C. Firing at 1200 °C produces a markedly denser and more continuous matrix (Fig. 8b, d and f), with substantially fewer interparticle voids than at 900 °C. The estimated pore-area fraction falls to 15–20% for CS0-1200, to 12–18% for CS5-1200 and to 10–15% for CS10-1200, *i.e.* by roughly 40–60% relative to the 900 °C specimen of the same composition. This concurrent decrease in pore-area fraction and increase in pore size reflects pore coalescence and closure associated with liquid-phase-assisted sintering. The microstructural changes are particularly evident for the CS containing specimens. CS5-1200 exhibits a dense continuous matrix with only isolated pores and the lowest visible coarse-pore population within the 1200 °C series, while CS10-1200 also shows pronounced matrix consolidation and the lowest estimated pore-area fraction of all specimens at 10–15%, although residual particles and local defects remain visible, including a bright residual particle of about 25–30 µm surrounded by matrix irregularities. These observations support the bulk-density and water-absorption results, indicating that the improved properties at high firing temperature are closely associated with enhanced matrix densification and reduced accessible porosity. The stronger densification observed in the CS containing ceramics at 1200 °C is consistent with the high-temperature behaviour of Fe- and Si-rich CS reported in previous studies. CS can participate in the formation of low-melting and glassy constituents, thereby promoting liquid-phase-assisted sintering and facilitating particle rearrangement and pore elimination.^26,41^ Similar development of compact matrices with increasing firing temperature has been reported for CS containing ceramic systems.^27^

**Fig. 8:**
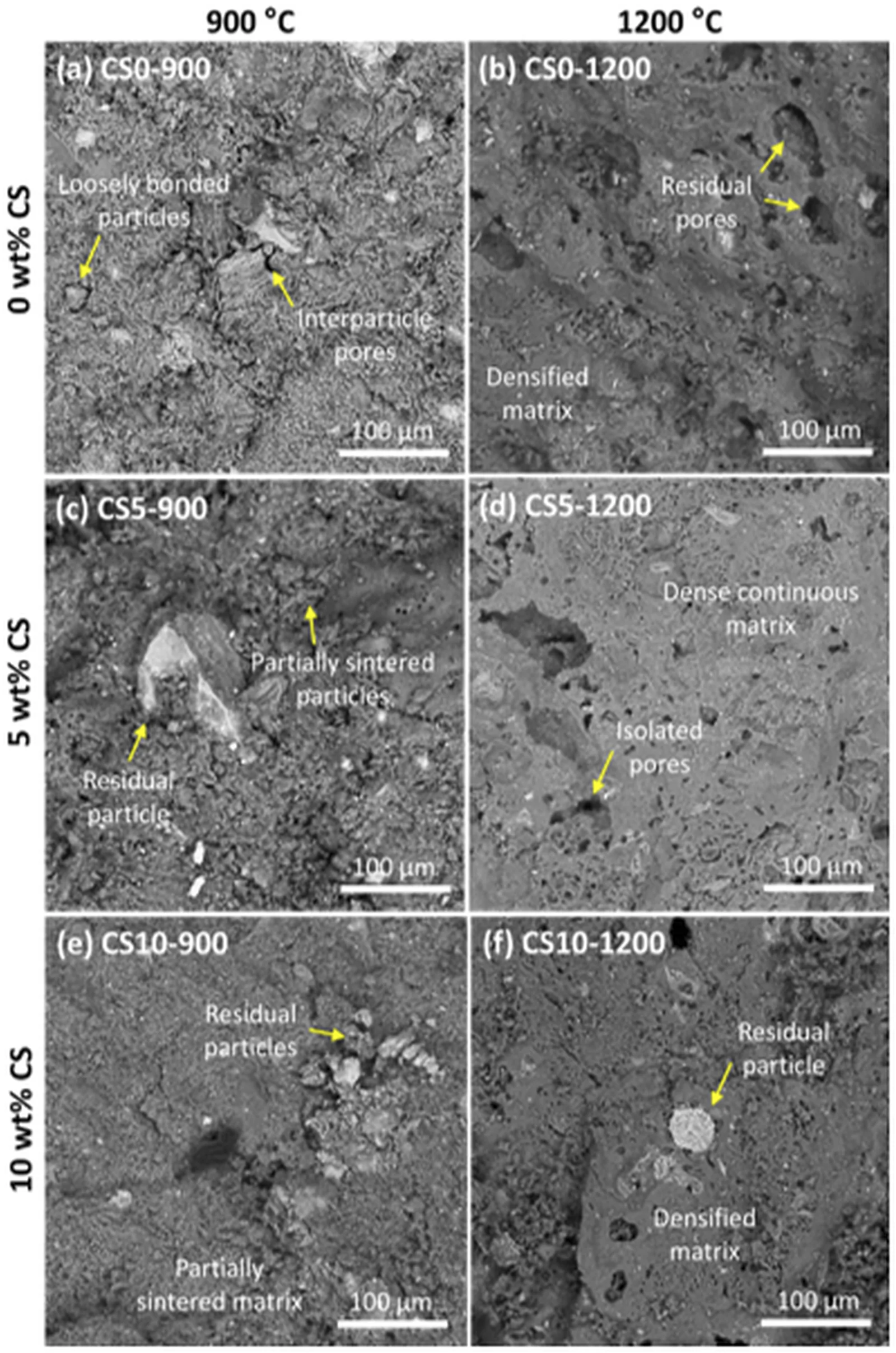
Fracture surface micrographs of ceramic samples with different CS contents after firing at 900 and 1200 °C: (a) CS0-900; (b) CS0-1200; (c) CS5-900; (d) CS5-1200; (e) CS10-900; and (f) CS10-1200.

### 3.3 Leaching behavior

The leaching behavior of the ceramics was evaluated using the TCLP method, and the concentrations of potentially hazardous elements in the leachates are summarized in Table 4. Despite the presence of potentially hazardous elements in CS, its incorporation at 5 and 10 wt% did not result in a systematic increase in their release from the fired ceramics. For the elements regulated under 40 CFR Part 261.24,^52^ the measured concentrations remained well below the corresponding toxicity characteristic limits. The leaching response was dependent on both the element and firing temperature. At 900 °C, increasing CS content resulted in higher Mn and, to a lesser extent, Cu concentrations, whereas no systematic increase was observed for Zn, As, Cd, or Pb. Firing at 1200 °C markedly reduced Cr and Mn release from the CS-containing ceramics. For CS10, Mn decreased from 540 to 44 µg L^−1^. In contrast, Cu release increased with firing temperature, reaching 76 µg L^−1^ for CS10-1200, while As reached 30 µg L^−1^. Notably, Pb release at 1200 °C decreased from 93 µg L^−1^ for CS0 to 27 µg L^−1^ for CS10, while Zn release remained comparable to or lower than that of the CS-free ceramic. The generally low leachability of the CS containing ceramics indicates effective retention of potentially hazardous elements within the fired matrix. The pronounced densification observed at 1200 °C may restrict their release by reducing accessible transport pathways, while incorporation into crystalline and glassy constituents may provide additional stabilization. Similar immobilization of potentially hazardous elements during high-temperature processing of CS containing ceramics has been reported previously.^27,30^ Nevertheless, the element specific trends, particularly for Cu and As, indicate that leaching behavior is not governed by densification alone and may also depend on the chemical state and phase distribution of individual elements. In this respect, the behaviour observed here is consistent with previously reported data for vitrified and fired slag bearing systems. Vitrification is known to reduce the leaching of Cd, Cr and Pb while increasing that of Cu and Zn, because Cu^2+^ and Zn^2+^ cannot substitute for Si^4+^ in the silicate framework and remain as charge balancing cations or oxides in the network interstices, where they are exchangeable with H^+^ in the acetic acid TCLP medium.^53^ For As, the effect was considerably weaker and not systematic. The behaviour of arsenic may depend on the structural rearrangement of SiO_2_ and the associated change in its binding with CaO during firing, as well as on the efficiency with which [AsO_4_] tetrahedra are incorporated into the silicate phases of the fired matrix. These aspects require further investigation. These results demonstrate low leachability of potentially hazardous elements from ceramics containing up to 10 wt% CS under the investigated TCLP conditions and indicate that the incorporation of up to 10% untreated CS can improve the engineering performance of the ceramics without causing a pronounced increase in leaching.

**Table 4:** TCLP leaching concentrations of ceramic specimens fired at 900 and 1200 °C and total hazaroads elements content in ceramics

Sample	Cr	Mn	Ni	Cu	Zn	As	Cd	Pb
**TCLP leaching concentrations, µg L** ^**−1**^								
Regulatory limits, CFR section 261.24	—	—	—	—	50	10	50	50
					00	00	00	00
CS0-900	13	250	<4.0	4	20	<10	63	42
CS5-900	10	390	<4.0	5	<1.3	20	<0.2	35
CS10-900	15	540	4.8	9	<1.3	14	3.8	34
CS0-1200	4.7	52	7.8	48	130	11	23	93
CS5-1200	4.9	56	4.7	46	65	16	2.4	42
CS10-1200	4.7	44	<4.0	76	100	30	2.9	27

**Total hazardous elements content, mg kg** ^**−1**^								
CS0	27	11	7.3	<5.0	26	9.5	2.5	35
CS5	46	135	8.8	140	160	20	2.7	63
CS10	66	260	10	270	290	31	2.9	92

Beyond the immobilization of potentially hazardous elements within the fired matrix, clay-derived and waste-derived ceramics are also actively explored as functional materials for water treatment.^54–57^ These applications rely on controlled porosity and permeability, which are governed by the same microstructural characteristics that determine water absorption and leachability in the present ceramics. This suggests that CS-containing ceramics may offer a broader functional potential beyond structural applications, although this direction requires separate investigation.

## 4 Conclusions

This study demonstrates the feasibility of incorporating untreated copper slag at relatively low contents (5–10 wt%) into waste-derived ceramic materials and reveals a pronounced temperature-dependent effect of CS on their performance. At 900–1000 °C, CS provided no clear mechanical benefit, whereas its contribution became increasingly pronounced at 1100–1200 °C. The most favorable combination of properties was achieved with 10 wt% CS at 1200 °C, reaching a compressive strength of approximately 135 MPa and a bulk density of 2.23 g cm^−3^, together with a low water absorption of approximately 2.2%.

The improvement in engineering properties at elevated temperatures was accompanied by substantial structural and microstructural evolution. XRD showed that the major crystalline phase assemblages of the CS-containing and reference ceramics remained broadly similar, indicating that the enhanced performance cannot be explained solely by changes in the major crystalline phases. FTIR revealed changes in the local silicate/aluminosilicate environment, while SEM confirmed pronounced consolidation and formation of a denser and more continuous matrix at 1200 °C. These results indicate that the beneficial high-temperature role of CS is likely associated with structural reorganization and enhanced densification of the ceramic matrix, possibly through liquid-phase-assisted sintering.

The incorporation of CS did not result in a systematic increase in the release of potentially hazardous elements. At 1200 °C, Pb leaching decreased from 93 µg L^−1^ for the CS free ceramic to 27 µg L^−1^ for CS10, while Zn release remained comparable to or lower than that of the reference material. Even for Cu and As, which showed some increase for CS10 at 1200 °C, the concentrations remained low, reaching only 76 and 30 µg L^−1^, respectively. For the elements regulated under 40 CFR Part 261.24, the measured concentrations remained well below the corresponding toxicity characteristic limits. These results indicate that the incorporation of up to 10 wt% untreated CS can improve the engineering performance of the ceramics without causing a pronounced increase in leaching under the investigated TCLP conditions. Thus, the proposed approach shows potential for the valorization of copper smelting slag in ceramic materials.

Finally, the role of CS should be considered within the combined effect of the ternary waste blend, and further tuning of the SS/FA ratio may provide additional room for optimization. Future work should focus on the long term durability and environmental performance of CS-containing ceramics under real service conditions, including freeze–thaw cycling, weathering, and extended leaching tests, as well as on assessing the scalability of the proposed formulations for industrial production.

## Author contributions

Dias Tolegenov: investigation, data curation, formal analysis, visualization, writing – review & editing. Madeniyet Yelubay: conceptualization, methodology, investigation, data curation, formal analysis, validation, visualization, writing – original draft, writing – review & editing. Sabit Maussumbayev: conceptualization, methodology, formal analysis, investigation, resources, software, validation, visualization, supervision, project administration, writing – original draft, writing – review & editing. Nurdana Kanasheva: methodology, resources, software, validation, writing – review & editing. Gulzat Aitkaliyeva: investigation, data curation, resources, validation, writing – review & editing. Tatyana Vakalova: methodology, resources, validation, writing – review & editing. Vladimir Mokichev: methodology, formal analysis, software, validation, writing – review & editing. Sergey Volynkin: formal analysis, software, validation, visualization, writing – review & editing. Anton Kasprzhitskii: conceptualization, methodology, supervision, validation, writing – review & editing. Georgy Lazorenko: conceptualization, supervision, project administration, resources, validation, writing – review & editing.

## Conflicts of interest

There are no conflicts to declare.

## Data Availability

The data supporting the findings of this study are included within the article.
